# EEG Signal Diversity Varies With Sleep Stage and Aspects of Dream Experience

**DOI:** 10.3389/fpsyg.2021.655884

**Published:** 2021-04-23

**Authors:** Arnfinn Aamodt, André Sevenius Nilsen, Benjamin Thürer, Fatemeh Hasanzadeh Moghadam, Nils Kauppi, Bjørn Erik Juel, Johan Frederik Storm

**Affiliations:** Brain Signalling Lab, Division of Physiology, Institute of Basic Medical Sciences, Faculty of Medicine, University of Oslo, Oslo, Norway

**Keywords:** EEG, signal diversity, complexity, entropy, sleep, dream, experience, consciousness

## Abstract

Several theories link consciousness to complex cortical dynamics, as suggested by comparison of brain signal diversity between conscious states and states where consciousness is lost or reduced. In particular, Lempel-Ziv complexity, amplitude coalition entropy and synchrony coalition entropy distinguish wakefulness and REM sleep from deep sleep and anesthesia, and are elevated in psychedelic states, reported to increase the range and vividness of conscious contents. Some studies have even found correlations between complexity measures and facets of self-reported experience. As suggested by integrated information theory and the entropic brain hypothesis, measures of differentiation and signal diversity may therefore be measurable correlates of consciousness and phenomenological richness. Inspired by these ideas, we tested three hypotheses about EEG signal diversity related to sleep and dreaming. First, diversity should decrease with successively deeper stages of non-REM sleep. Second, signal diversity within the same sleep stage should be higher for periods of dreaming vs. non-dreaming. Third, specific aspects of dream contents should correlate with signal diversity in corresponding cortical regions. We employed a repeated awakening paradigm in sleep deprived healthy volunteers, with immediate dream report and rating of dream content along a thought-perceptual axis, from exclusively thought-like to exclusively perceptual. Generalized linear mixed models were used to assess how signal diversity varied with sleep stage, dreaming and thought-perceptual rating. Signal diversity decreased with sleep depth, but was not significantly different between dreaming and non-dreaming, even though there was a significant positive correlation between Lempel-Ziv complexity of EEG recorded over the posterior cortex and thought-perceptual ratings of dream contents.

## Introduction

While there is still no universally accepted theory of consciousness, or full consensus on what the neural correlates of consciousness may be, there seems to be some points of approximate convergence. Many researchers and models of consciousness have (to varying degree) emphasized the informative and integrated^1^ aspects of conscious experience (Baars, 1988; Crick and Koch, 1990; Chalmers, 1995; Llinás et al., 1998; Tononi and Edelman, 1998; Dehaene and Naccache, 2001; Edelman, 2003; Mashour, 2004; Seth et al., 2005; Barrett, 2014; Carhart-Harris et al., 2014; Ruffini, 2017; Mashour et al., 2020), a point that has perhaps been most clearly expressed and developed in Tononi's Integrated Information Theory (IIT) (Tononi, 2004; Oizumi et al., 2014). In line with this idea, it is widely believed that conscious experience depends on (i.e., supervenes on) complex neural dynamics in richly interconnected thalamocortical networks (Tononi and Edelman, 1998; Dehaene and Naccache, 2001; Seth et al., 2005; Buzsáki, 2007; Baars et al., 2013; Tononi et al., 2016) [but see Merker (2007, 2013) for an account emphasizing subcortical structures]. Over the last couple of decades, this hypothesis has received substantial empirical support (Shaw et al., 1999; Massimini et al., 2005; Casali et al., 2013; Sitt et al., 2014; Casarotto et al., 2016; Schartner, 2017; Mashour and Hudetz, 2018). Not only do measures of complexity, differentiation and/or integration decrease from wakefulness to states where conscious experience is severely impoverished or completely lost, and increase in psychedelic states, there is also emerging evidence that such measures may correlate with ratings of aspects of phenomenology within the same state (Boly et al., 2015; Schartner et al., 2017a; Timmermann et al., 2019; Farnes et al., 2020).

In terms of reliable stratification of different states and conditions according to their clinically or conventionally ascribed level of consciousness,^2^ the perturbational complexity index (PCI), which captures elements of both integration and differentiation, is perhaps the most impressive of the proposed measures of consciousness (Casali et al., 2013; Sarasso et al., 2015; Casarotto et al., 2016). However, PCI is based on the complexity of the average, source-reconstructed cortical response to hundreds of targeted electromagnetic pulses, usually delivered by transcranial magnetic stimulation (TMS), making it technically more demanding than indicators calculated directly from spontaneous brain signals [but see Comolatti et al. (2019)]. Motivated by this, Schartner et al. applied measures of complexity and entropy to spontaneous multichannel brain signals, suggesting that Lempel-Ziv complexity (LZC), amplitude coalition entropy (ACE) and synchrony coalition entropy (SCE) may serve as indicators of consciousness (Schartner et al., 2015; Schartner, 2017). While these diversity measures do not (directly) capture cortical integration, they have nevertheless shown promise in distinguishing states associated with conscious experience, such as wakefulness and REM sleep, from states were conscious experience is degraded or possibly lost, such as slow wave sleep and propofol anesthesia (Schartner et al., 2015, 2017b). In particular, stereo-EEG signal diversity from ten epileptic patients was shown to be higher in wakefulness and REM sleep than NREM3 sleep (with intermediate values for NREM2, in a single patient for which data was available) (Schartner et al., 2017b). Single channel LZC has also been shown to decrease with sleep depth in humans (Andrillon et al., 2016), and to be higher in wakefulness and REM sleep than non-REM sleep in rats (Abásolo et al., 2015), in line with evidence from studies using alternative measures of (temporal) complexity and entropy (Ma et al., 2018).

Intriguingly, MEG and EEG signal diversity was elevated in psychedelic states induced by LSD, psilocybin, DMT, and subanesthetic doses of ketamine, in line with reports of increased range and intensity of conscious contents in psychedelic states, and has even been found to correlate with some subjective ratings of psychedelic phenomenology (Schartner et al., 2017a; Timmermann et al., 2019; Farnes et al., 2020). Hence, signal diversity (of a system) may be a correlate of consciousness and richness of subjective experience (supported by that system), at least under certain conditions and within a limited range, as suggested by complexity theories such as IIT and Carhart-Harris' speculative entropic brain hypothesis (Marshall et al., 2016; Tononi et al., 2016; Carhart-Harris, 2018) [but see Papo (2016)].

Inspired by this general idea,^3^ we here derive and test three specific hypotheses about how multi-channel EEG signal diversity relates to sleep and dreaming.^4^ First, we hypothesize that signal diversity should decrease with successively deeper stages of NREM sleep, in line with reported incidence of dreaming across sleep stages, and subjective ratings of dream experience richness (Siclari et al., 2013). Second, within the same sleep stage (NREM2), signal diversity should be higher before awakenings with reported dream experience (DE) vs. non-experience (NE).^5^ Third, the degree to which specific dimensions of conscious content dominate the dream experience should correlate with signal diversity in corresponding cortical regions. Particularly, we suggest that signal diversity in posterior cortical areas should correlate with higher ratings of dream experience on a thought-perceptual scale, while frontal signal diversity should be negatively (or less strongly) correlated to thought-perceptual ratings (Siclari et al., 2013, 2017).

## Methods

The Regional Committees for Medical and Health Research Ethics approved this research project (ref. 2018/1640), and informed consent was collected before participation.

We employed a sleep study paradigm with serial awakenings and dream reports, similar to Siclari et al. (2013), but our experiments were performed in the morning, after a night of sleep deprivation. Participants were instructed to avoid caffeine in the afternoon the day before the experiment, to stay awake through the whole night, and to meet in the lab at 7 a.m. Before the experiment started, participants received an explanation of the questions they would be asked when awakened by the alarm. Experimenters stayed in the room throughout the experiment to monitor the EEG recording. Background sounds were masked by brown noise played through ear plugs in the participant's ears. The participant was intermittently awakened by an electronic alarm and immediately asked about any dream experience they might have had right before the alarm sound, by the prompt: “What was the last thing going through your mind?”. Awakenings were classified as no experience (NE), dream experience without recall (DEWR), or dream experience (DE). If the participant reported experiencing something, they were also asked to rate their experience on a five-point scale from exclusively thought-like (thinking or reasoning, with no sensory content) to exclusively perceptual (vivid sensory content, without thinking or reasoning) (Siclari et al., 2013). To optimize the balance of NE and DE awakenings for within-state analysis, awakenings were mainly performed during NREM2 sleep.

We recruited 17 healthy, non-smoking participants between 18 and 35 years, with normal sleep habits. One participant was excluded from the analysis due to inability to sleep during the experiment. For each of the 16 remaining participants, we recorded EEG from multiple sleep trials, defined as the period from the participant was told that they could go (back) to sleep, until they were (again) awakened by the alarm, for a total of 97 sleep trials. For each sleep trial, we marked non-overlapping sleep epochs, defined as 30 s segments of EEG, aligned to the time of awakening. We scored the sleep stage of the last ten sleep epochs before awakening according to the AASM Manual for Scoring of Sleep and Associated Events (Berri et al., 2018), for a total of 960^6^ scored sleep epochs (wake = 72, NREM1 = 206, NREM2 = 619, NREM3 = 53, REM = 10). There were 62 awakenings (NE = 9, DEWR = 9, DE = 41, unclear/missing report = 3) from NREM2 sleep, which were used for within-state analysis of whether signal diversity was different for dreaming vs. non-dreaming. Of these, only the 41 reports of dream experiences (exclusively thought-like = 3, mostly thought-like = 11, equally perceptual and thought-like = 10, mostly perceptual = 7, exclusively perceptual = 8, unclear/missing report = 2) from NREM2 sleep were included in the analysis of the thought-perceptual dimension of dream content.

EEG was sampled at 1,000 Hz, using two 32 channel amplifiers to record 62 EEG channels and two electrooculogram (EOG) channels (BrainAmp DC, Brain Products GmbH, Gilching, Germany). The reference and ground electrodes were placed medially on the forehead. Electromyogram (EMG) was recorded by two bipolar electrodes on the chin.

Data was processed using MNE Python and EEGLAB (Delorme and Makeig, 2004; Gramfort et al., 2013). After sleep scoring and marking of bad segments, datasets were automatically preprocessed using the EEG-Clean-Tools EEGLAB-plugin (Bigdely-Shamlo et al., 2015). Independent component analysis (ICA) was performed using the extended infomax algorithm (Bell and Sejnowski, 1995), and bad components were rejected by heuristic rules based on the source probabilities assigned by the IClabel-plugin (Pion-Tonachini et al., 2019). After band-pass filtering (0.75–40 Hz), the data was Laplace transformed by a spherical spline algorithm (Perrin et al., 1989) implemented in the CSD Toolbox (Kayser and Tenke, 2006; Fitzgibbon et al., 2015), and imported back into Python.

**Figure 1:**
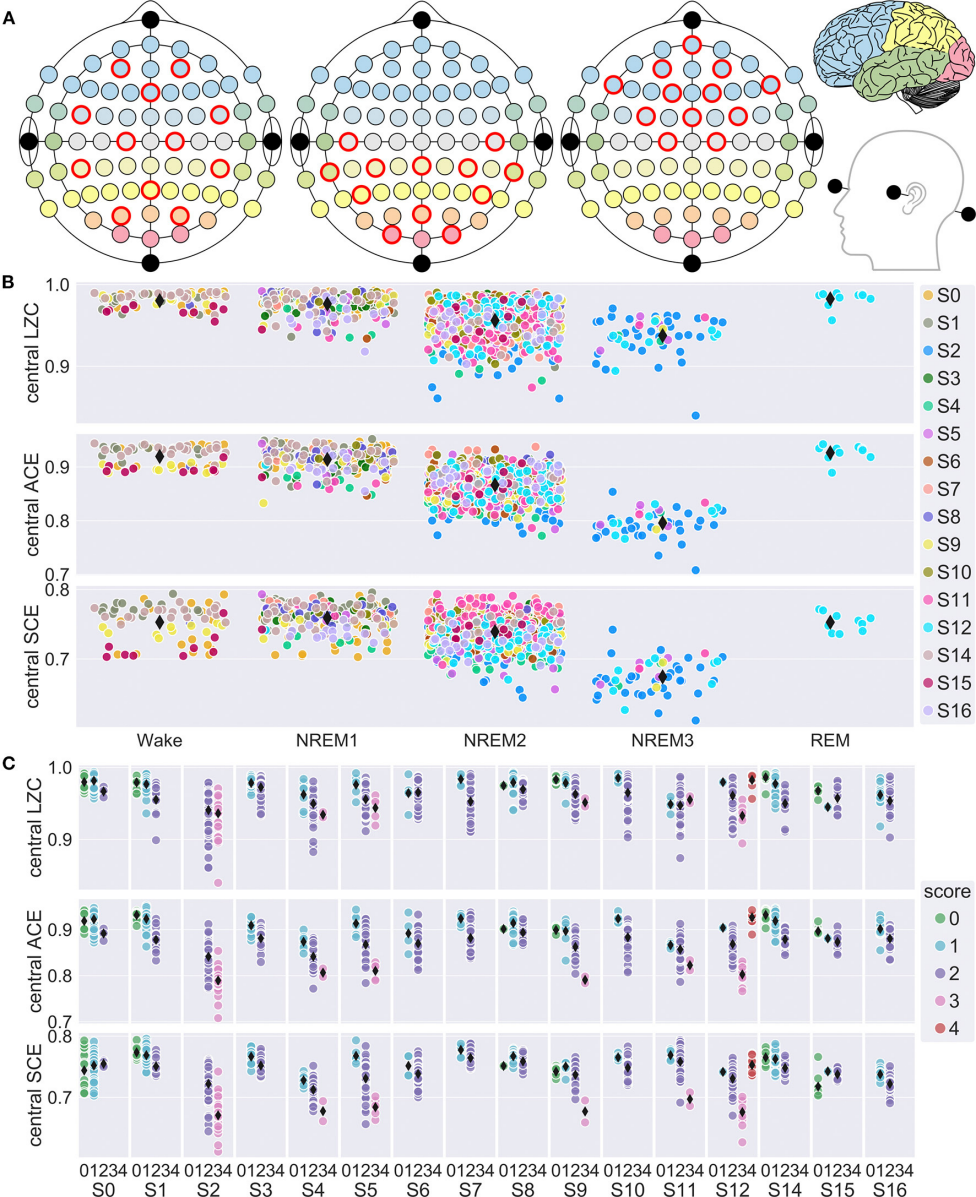
EEG channel selection and variation in signal diversity with sleep stage. **(A)** Central, posterior and frontal channel selections. Electrode fill color indicates associated cortical lobe [adapted from illustration by Laurens R. Krol, distributed under a CC0 1.0 license (Krol, 2020)]. **(B)** Mean LZC, ACE and SCE for all 30 s sleep epochs vs. sleep stage for the central channel selection. Observations are randomly jittered along x-axis to reduce overlap, and participant number (S0, …, S16) is indicated by marker fill color. Grand mean values for each sleep stage is indicated by black diamond markers. Most of the data is from NREM2 sleep, because we prioritized the within-state analysis of dream-reports from NREM2. In particular, all REM sleep epochs are from a single sleep trial for Participant 12. **(C)** Mean central LZC, ACE and SCE for the 30 s sleep epochs vs. sleep stage (0 = W, 1 = NREM1, 2 = NREM2, 3 = NREM3, 4 = REM), plotted separately for each study participant (S0, …, S16). Fill color indicates sleep stage, and diamond markers indicates participant mean values for each stage.

EEG was divided into regular 8 s windows with 7 s overlap. LZC, ACE and SCE was calculated for each 8 s window (Schartner et al., 2015), using a selection of 12 EEG channels (AF3, AF4, FC5, Fz, FC6, C1, C2, CP5, Pz, CP6, PO3, PO4) covering large parts of the scalp (Figure 1A), and then averaged over each 30 s sleep epoch. The calculation was repeated for a selection of 12 frontal channels (AF3, Fpz, AF4, F7, F1, F2, F8, FC3, FCz, FC4, C1, C2) and a selection of 12 posterior channels (C5, CPz, C6, TP7, CP3, CP4, TP8, P5, POz, P6, O1, O2).

All three diversity measures are based on the complex valued analytic representation of the recorded EEG signal. Before calculating LZC, the analytic signal for each window was binarized by thresholding on the single-channel median absolute value. All the observations in an window were concatenated together timepoint by timepoint to form one long binary string. The LZC algorithm itself can be viewed as a way to compress such a binary string into a shorter description, by exploiting repeating patterns. The algorithm works by iteratively building a dictionary of unique substrings, that allows reconstruction of the full string. The length of the resulting dictionary serves as a measure of how complex the original string is. Normalizing by the dictionary length for a randomly permuted version of the original binary string, yields a LZC value between zero and one (almost always).

ACE used the same binarization of the analytical signal as LZC, but we did not concatenate observations together. Instead, the 12 channel binary vectors for each timepoint, which we may call *amplitude coalitions*, are themselves considered states. Within each window, we approximate the probability distribution of these states by the observed incidence of each amplitude coalition. The ACE is the Shannon entropy of this estimated probability distribution, normalized by the entropy for a randomly permuted copy of the original binary matrix.

For SCE, we (iteratively) choose a single reference channel, and we binarize each of the remaining 11 channels by thresholding on the absolute phase difference of their analytic signal relative to the current reference channel (at ~45°). The 11 channel binary vectors for each timepoint may be considered *synchrony coalitions* of the system, describing the state of the system in terms of which channels are currently in phase with the reference channel. Just like for ACE, we can estimate the probability distribution for the synchrony coalitions (relative to the current reference), and calculate the normalized Shannon entropy based on this distribution. The overall SCE is then obtained by averaging over the 12 different choices of reference channel. For further information about the calculation of LCZ, ACE and SCE, see (Schartner, 2017).

Statistical analysis was performed using IBM SPSS version 26. The relationship between sleep stage and signal diversity was assessed using generalized linear mixed models (GLMMs) with sleep stage as a fixed factor^7^ (including intercept), and participant and trial (nested within participants) as random intercepts. Epochs were entered as repeated measures with heterogeneous first-order autoregressive residual variance-covariance.

For awakenings in NREM2 sleep, the relationship between experience report (NE, DEWR or DE) and signal diversity of the last sleep epoch before awakening was assessed using GLMMs with experience classification as the fixed factor (including intercept) and subject as random intercept.

Finally, for NREM2 awakenings with reported dream experience (DE), the correlation between subjective ratings of experience on a five-point scale from exclusively thought-like to exclusively perceptual, and frontal and posterior signal diversity were assessed using GLMMs with thought-perceptual rating as a fixed continuous covariate (including intercept) and subject as random intercept.

We used the inheritance procedure (Goeman and Finos, 2012) to adjust for multiple comparisons and control the family-wise error rate. Starting with significance level α = 0.05, this yielded an adjusted significance level α/12 = 0.0042 for the effect of sleep stage, experience classification and thought-perceptual rating on signal diversity, and α/(12·10) = 0.00042 for each of the 10 pairwise comparisons between different sleep stages.

For further details, see Supplementary Material.

## Results

First, we analyzed variation in signal diversity with sleep stage. Figures 1B,C show overall and individual trends. Results of the mixed model analysis are summarized in Table 1. Sleep stage was a significant factor in the model for both LZC [*F*_(4,139)_ = 32.5, *p* < 0.0001], ACE [*F*_(4,169)_ = 67.7, *p* < 0.0001] and SCE [*F*_(4,235)_ = 37.4.0, *p* < 0.0001]. Pairwise comparisons of estimated marginal means indicated that diversity for NREM3 sleep was lower than for NREM2, and diversity for NREM2 was lower than for wake and NREM1 sleep, but the difference in LZC between stages NREM2 and NREM3 was no longer significant after adjusting for multiple comparisons. Estimated differences in mean signal diversity between wake and NREM3 sleep (Δ_LZC_ = 0.029, Δ_ACE_ = 0.078, Δ_SCE_ = 0.044) were around 3–4 times the estimated between-participant standard deviations (s_LZC_ = 0.0069, s_ACE_ = 0.0173, s_SCE_ = 0.0141). Estimates for the *participant* × *trial* random effect were similar (s_LZC_ = 0.0066, s_ACE_ = 0.0100, s_SCE_ = 0.0141). The contrast between wakefulness and NREM1 sleep was not significant. This data set only included a single trial of REM sleep, because we were mostly interested in variation in signal diversity with depth of non-REM sleep, and within-state analysis of dreaming in NREM2 sleep. However, estimated marginal mean signal diversity was mostly higher for REM sleep than for NREM2 and NREM3 (but the estimated difference in SCE between REM and NREM2 sleep was not significant). Estimated mean ACE was also higher for REM than for stages W and NREM1. Only the LZC REM-NREM3 contrast, the ACE REM-NREM2 and the ACE REM-NREM3 contrasts remained significant after multiple comparison correction.

**Table 1:** Summary of results.

**Response**	**Fixed effect**	**df1**	**df2**	***F***	**sig**.					
**LZC**	*Sleep stage*	4	139	32.5	<0.0001					
							**sig. (pairwise comparison)**			
	**EMMs**	**Estimate**	**std. err**.	**CI**_**LOWER**_	**CI**_**UPPER**_		*NREM1*	*NREM2*	*NREM3*	*REM*
	*Wake*	0.976	0.002	0.971	0.981		0.2068	<0.0001	<0.0001	0.5575
	*NREM1*	0.974	0.002	0.970	0.979			<0.0001	<0.0001	0.4016
	*NREM2*	0.959	0.002	0.955	0.963				0.0049	0.0065
	*NREM3*	0.947	0.005	0.937	0.956					0.0002
	*REM*	0.981	0.008	0.965	0.997					
**Response**	**Fixed effect**	**df1**	**df2**	***F***	**sig**.					
**ACE**	*Sleep stage*	4	169	67.7	<0.0001					
							**sig. (pairwise comparison)**			
	**EMMs**	**Estimate**	**std. err**.	**CI**_**LOWER**_	**CI**_**UPPER**_		*NREM1*	*NREM2*	*NREM3*	*REM*
	*Wake*	0.906	0.005	0.896	0.916		0.1295	<0.0001	<0.0001	0.0491
	*NREM1*	0.902	0.005	0.893	0.912			<0.0001	<0.0001	0.0228
	*NREM2*	0.873	0.004	0.864	0.883				<0.0001	<0.0001
	*NREM3*	0.828	0.007	0.814	0.843					<0.0001
	*REM*	0.932	0.013	0.906	0.959					
**Response**	**Fixed effect**	**df1**	**df2**	***F***	**sig**.					
**SCE**	*Sleep stage*	4	235	37.4	<0.0001					
							**sig. (pairwise comparison)**			
	**EMMs**	**Estimate**	**std. err**.	**CI**_**LOWER**_	**CI**_**UPPER**_		*NREM1*	*NREM2*	*NREM3*	*REM*
	*Wake*	0.755	0.005	0.745	0.765		0.1163	<0.0001	<0.0001	0.5363
	*NREM1*	0.751	0.004	0.742	0.760			<0.0001	<0.0001	0.3901
	*NREM2*	0.740	0.004	0.731	0.749				<0.0001	0.1111
	*NREM3*	0.711	0.005	0.700	0.721					0.0008
	*REM*	0.765	0.016	0.734	0.796					
**Response**	**Fixed effect**	**df1**	**df2**	***F***	**sig**.					
**LZC**^a^	*Experience class*	2	40	0.516	0.6009					
							**sig. (pairwise comparison)**			
	**EMMs**	**Estimate**	**std. err**.	**CI**_**LOWER**_	**CI**_**UPPER**_			*DEWR*	*DE*	
	*NE*	0.956	0.007	0.941	0.971			0.6889	0.6679	
	*DEWR*	0.960	0.007	0.947	0.974				0.3266	
	*DE*	0.953	0.004	0.945	0.960					
**Response**	**Fixed effect**	**df1**	**df2**	***F***	**sig**.					
**ACE**	*Experience class*	2	56	0.254	0.7766					
							**sig. (pairwise comparison)**			
	**EMMs**	**Estimate**	**std. err**.	**CI**_**LOWER**_	**CI**_**UPPER**_			*DEWR*	*DE*	
	*NE*	0.866	0.011	0.843	0.889			0.4808	0.6535	
	*DEWR*	0.856	0.010	0.835	0.877				0.6362	
	*DE*	0.861	0.006	0.848	0.875					
**Response**	**Fixed effect**	**df1**	**df2**	***F***	**sig**.					
**SCE**	*Experience class*	2	54	1.03	0.3626					
							**sig. (pairwise comparison)**			
	**EMMs**	**Estimate**	**std. err**.	**CI**_**LOWER**_	**CI**_**UPPER**_			*DEWR*	*DE*	
	*NE*	0.725	0.008	0.708	0.741			0.6944	0.2224	
	*DEWR*	0.728	0.007	0.714	0.743				0.3764	
	*DE*	0.734	0.005	0.724	0.745					
**Response**	**Fixed effect**	**df1**	**df2**	***F***	**sig**.	**Estimate**	**std. err**.	**t**	**CI**_**LOWER**_	**CI**_**UPPER**_
**LZC**_**BACK**_	*Thought-percept*	1	34	9.96	0.0033	0.0076	0.0024	3.16	0.0027	0.0124
**LZC**_**FRONT**_	*Thought-percept*	1	31	4.45	0.0432	0.0067	0.0032	2.11	0.0002	0.0132
**ACE**_**BACK**_	*Thought-percept*	1	32	4.27	0.0472	0.0074	0.0036	2.07	9.89e-5	0.0147
**ACE**_**FRONT**_	*Thought-percept*	1	29	2.31	0.1398	0.0055	0.0036	1.52	0.0019	0.0128
**SCE**_**BACK**_	*Thought-percept*	1	34	0.021	0.8864	0.0004	0.0029	0.144	0.0055	0.0063
**SCE**_**FRONT**_	*Thought-percept*	1	29	0.370	0.5478	−0.0016	0.0026	−0.608	−0.0070	0.0038

*Signal diversity (back transformed from identity link gamma GLMM for 1–SD) as a function of sleep stage, experience class and thought-perceptual rating of dreams*.

a *The CI for the between-participant variance in this model was gigantic, indicating possible problems with model fitting. Excluding/winsorizing the smallest one/two LZC values lead to plausible CI, and gave otherwise similar results, as did a beta GLMM with logit link (Supplementary Material). NE, no experience; DEWR, dream experience without recall; DE, dream experience*.

**Figure 2:**
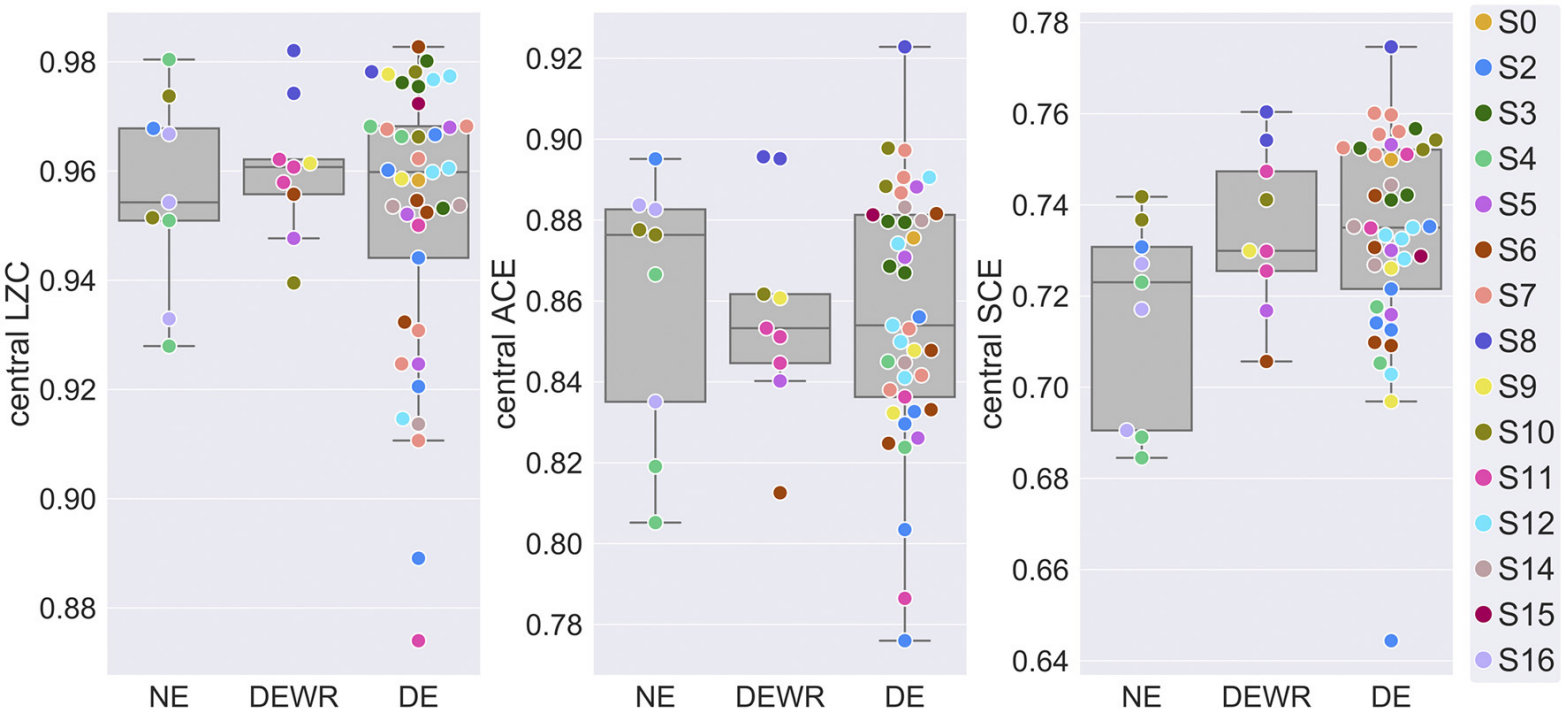
EEG signal diversity vs. dream experience classification. Mean central LZC, ACE and SCE of the last 30 s sleep epoch before NREM2 awakenings, vs. experience classification of subsequent dream reports (DE = dream experience, DEWR = dream experience without recall of contents, NE = non-experience). Observations plotted on top of corresponding boxplots. Participant number (S0, …, S16) is indicated by marker fill color, and observations are displaced slightly along x-axis to avoid overlap.

Next, we analyzed whether experience classification was a significant factor in models of signal diversity within NREM2 sleep. Figure 2 shows signal diversity vs. experience classification for the last 30 s sleep epoch before awakening and report. Results of the statistical analysis are summarized in Table 1 (but see footnote about possible model-fitting problems for the LZC model). Experience classification was not a significant factor in the models for LZC [*F*_(2,40)_ = 0.516, *p* = 0.601], ACE [*F*_(2,56)_ = 0.254, *p* = 0.777] or SCE [*F*_(2,54)_ = 1.03, *p* = 0.363]. Looking at the estimated marginal means, only SCE had monotonically increasing signal diversity going from no experience (NE) to dreaming (DE). The difference in SCE between DE and NE was just over half the estimated between-participant standard deviation. Given the ongoing debate about the extent to which (pre)frontal and posterior cortex directly contribute to the contents of conscious experience (see Discussion), we also re-ran the analysis with a posterior channel selection (Figure 1A), but experience classification was still not a significant factor (Supplementary Material).

**Figure 3:**
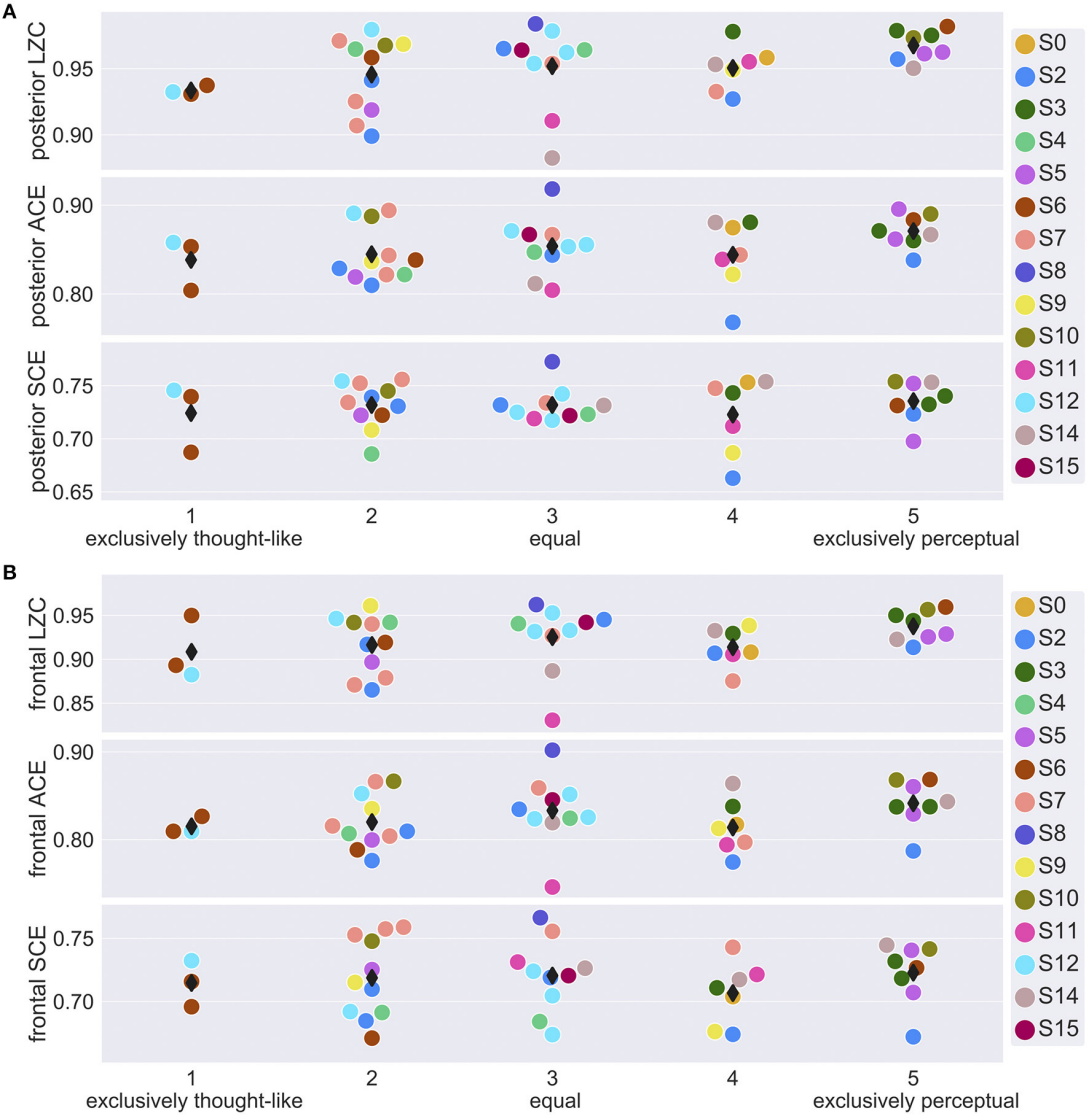
EEG signal diversity vs. thought-perceptual ratings of dream experience. **(A)** Mean posterior (see Figure 1A) signal diversity of the last 30 s sleep epoch before NREM2 awakenings with recalled dream experience, vs. thought-perceptual ratings of dream contents (1 = exclusively thought-like, 5 = exclusively perceptual). Participant number (S0, …, S16) is indicated by marker fill color, and observations are displaced slightly along x-axis to avoid overlap. **(B)** Mean frontal (see Figure 1A) signal diversity of the last 30 s sleep epoch before NREM2 awakenings with recalled dream experience, vs. thought-perceptual ratings of dream contents.

Finally, we explored whether signal diversity from a posterior and a frontal channel selection (Figure 1A) correlated with subjective ratings of dream experience on a thought-perceptual axis. Figure 3 shows posterior and frontal signal diversity vs. thought-perceptual ratings. Statistical results are summarized in Table 1. Thought-perceptual rating was a significant positive covariate of posterior LZC [*F*_(1,34)_ = 9.96, *p* = 0.0033] and ACE [*F*_(1,32)_ = 4.27, *p* = 0.0472], as well as frontal LZC [*F*_(1,31)_ = 4.45, *p* = 0.0432]. Estimated slope for these models (β_LZC,back_ = 0.0076, β_ACE,back_ = 0.0074, β_LZC,front_ = 0.0067) corresponded to an increase from “exclusively thought-like” to “exclusively perceptual” of about 1.5–3 times the estimated between-participant standard deviation (s_LZC,back_ = 0.010, s_ACE,back_ = 0.020, s_LZC,front_ = 0.0173). Estimated coefficients for thought-perceptual rating were slightly smaller (or more negative in the case of SCE) for frontal signal diversity, compared to posterior signal diversity. Only the correlation between thought-perceptual rating and posterior LZC remained significant after adjusting for multiple comparisons.

## Discussion

We found sleep stage to be a significant factor in determining signal diversity of scalp EEG, for all diversity measures used. The reductions in estimated marginal mean LZC, ACE and SCE between wake and NREM3 were large compared to between-participant variation, and for all three diversity measures, estimated marginal means became progressively lower going from wakefulness to NREM3, consistent with the hypothesis that signal diversity decreases with depth of NREM sleep. Our results thus support and extend the findings in Schartner et al. (2017b) from comparison of stereo-EEG signal diversity between wake, REM and NREM3 in epileptic patients, as well as previous studies indicating that single channel LZC varies with sleep depth in humans and animals (Shaw et al., 1999; Abásolo et al., 2015; Andrillon et al., 2016). Studies using TMS-EEG (Massimini et al., 2005; Casali et al., 2013), or different measures of (temporal) complexity and entropy, point in the same direction (Ma et al., 2018). However, sleep-cycle changes in (dispersion) entropy is time-scale dependent, and correlated to the slope of the power spectrum, both possibly reflecting excitatory-inhibitory balance (Miskovic et al., 2019). The decrease in signal diversity with increasing depth of NREM sleep does not necessarily reflect a (direct) relationship between signal diversity and richness of experience. It may instead (primarily) reflect physiological changes, e.g., in level of arousal, as potentially suggested by the widespread increases in fMRI brain entropy observed after administration of caffeine (Chang et al., 2018).

Conversely, experience classification was not a significant factor in the models of how LZC, ACE and SCE varies between awakenings within NREM2 sleep, and there were no significant contrasts between NE, DEWR and DE awakenings, even before correcting for multiple comparisons. Signal diversity was not consistently higher for DE than NE awakenings. However, there is an ongoing debate about the extent to which (pre-)frontal cortex (directly) supports conscious experience, with some evidence indicating that the neural correlates of consciousness may be mostly located in posterior cortex (Koch et al., 2016; Boly et al., 2017; Odegaard et al., 2017; Storm et al., 2017). In particular (Siclari et al., 2017), found that dream experience was associated with local decreases in 1–4 Hz activity in the posterior cortex relative to awakenings with no dream experience, and a later study found that a subset of large, steep slow waves in frontal cortical regions were associated with successful dream recall (Siclari et al., 2018). We therefore re-ran the analysis using a posterior channel selection, as a *post-hoc* test to see if that would have changed results. While estimated marginal means were always higher for DE than NE, differences between NE and DE were still small and far from statistical significance. It should be noted that the sample sizes for NE and DEWR awakenings were small, due to higher frequency of dreaming in NREM2 than expected. Furthermore, we cannot exclude the possibility that some reports may be inaccurate due to memory failure. In fact, some researchers consider the question of whether phenomenal consciousness is ever lost during sleep to be an open question (Windt et al., 2016). While the current result does not allow us to reject the null hypothesis that signal diversity is the same for NE and DE awakenings, other studies suggest that cortical bi-stability, posterior low-frequency power, and (posterior) cortical connectivity at low frequencies may differ between dreaming and non-dreaming (Nieminen et al., 2016; Siclari et al., 2017; Lee et al., 2019), although a recent study failed to classify dreaming from non-dreaming using EEG spectral power (Wong et al., 2020).

Thought-perceptual rating of NREM2 dream experiences was a positive covariate of both posterior and frontal signal diversity, except for frontal SCE, which was negatively related to thought-perceptual ratings. The coefficient estimates were significantly different from zero for posterior LZC and ACE, as well as frontal LZC, but only remained significant for posterior LZC after adjusting for multiple comparisons. This novel finding complements the previously reported positive correlation between thought-perceptual ratings and posterior high-frequency power in REM sleep (Siclari et al., 2017), and adds to emerging evidence that signal diversity may correlate with aspects of conscious contents (Schartner et al., 2017a; Timmermann et al., 2019; Farnes et al., 2020).

In interpreting the results of this study, one should keep in mind that there are many physiological changes between sleep stages, and it is unlikely that all of these directly relate to changes in conscious experience. Furthermore, exactly how richness of experience varies with sleep stage is not well-established. Hence, we cannot exclude alternative interpretations of the relationship between sleep stage and signal diversity, that do not require a link between signal diversity and richness of experience. Similarly, the significant correlation between thought-perceptual rating and posterior LZC could in principle be influenced by unmeasured confounding variables, since NREM2 sleep is not a monolithic state. As with any study of dream reports, we cannot guarantee that subjective reports are reliable. We tried to minimize the risk that unreliable reporting could impact our results by carefully instructing participants before the experiment, and by excluding from the analysis dream reports that were too unclear to be categorized. Finally, a small sample size, particularly for NE awakenings, is an important limitation of this study, as is the failure to collect subjective ratings of dream experience richness, which could have allowed a more direct test of the entropic brain hypothesis.

To what extent do signal diversity measures provide independent information relative to the information already contained in the EEG power spectrum? Previous studies suggest that brain signal entropy is correlated with the slope of the power spectrum (Miskovic et al., 2019), but that signal diversity measures nevertheless provides additional information beyond the spectral properties of the EEG signal (Schartner et al., 2015, 2017b). Plots of relative EEG power in different frequency bands (see Supplementary Figures 2–4) seem to suggest, as expected, that increased power in high frequency bands and decreased power in low frequency bands is associated with increased signal diversity. We have not analyzed this question in more detail in this small study, but the relationship between signal diversity and spectral properties should be further investigated in future studies.

Both between-states and within-state studies of measures of consciousness could be improved by simultaneous independent measurement of important covariates of brain activity and/or experience, e.g., arousal and/or apical drive (Aru et al., 2020). The discovered relationship between thought-perceptual ratings and posterior LZC should be confirmed in follow-up studies, both for NREM2 sleep and other states, such as waking imagination with eyes closed. This result could also conceivably be extended to more fine-grained categories of experience, relating (time domain) signal diversity of single channels or source reconstructed EEG in clearly delineated regions (e.g., fusiform face area) to specific percepts (e.g., faces) (Siclari et al., 2017). Within-state differences in signal diversity between experience and non-experience should be tested with larger sample sizes, and should be supplemented by more informative graded ratings of phenomenological richness. In particular, multi-dimensional ratings of experience (e.g., along dimensions of diversity and vividness) would give a multivariate description of richness of experience, to be approximately reproduced by candidate measures (say, spatial and temporal LZC).

Supporting and extending the results from previous studies, we found that signal diversity of scalp EEG in healthy volunteers changed significantly with sleep stage, and was progressively reduced with deeper stages of non-REM sleep. On the other hand, we failed to find any significant difference in signal diversity between NREM2 periods followed by dream reports and those that were not. We did, however, find a significant positive correlation between LZC in a channel selection covering the posterior cortex, and more perceptual ratings of dream content on a thought-perceptual scale. This novel result adds to a growing body of evidence suggesting a potential link between measures of neural differentiation and contents of experience.

Overall, our findings are consistent with the hypothesis that signal diversity is a correlate of phenomenological richness (or brain states that favor such richness), but results are somewhat mixed, and alternative interpretations cannot be ruled out. Therefore, further studies are needed, with larger sample sizes and explicit subjective rating of richness of experience.

## Data Availability Statement

The raw data supporting the conclusions of this article will be made available by the authors, without undue reservation.

## Ethics Statement

The studies involving human participants were reviewed and approved by Regional Committees for Medical and Health Research Ethics - South East, Faculty of Medicine, University of Oslo (ref. 2018/1640). The patients/participants provided their written informed consent to participate in this study.

## Author Contributions

AAa wrote the paper, interpreted the results, analyzed the data, and helped collect the data and design the study. AN revised the paper and helped interpret the results, plan the analysis, and collect the data and design the study. BT revised the paper, collected the data, and initiated and designed the study. FM revised the paper, collected the data, and helped design the study. NK revised the paper, and helped collect the data and design the study. BJ revised the paper and helped interpret the results, initiate and design the study. JS revised the paper, helped interpret the results, initiate and design the study, and started the Norwegian Research Council project which this study is a part of, and he is the leader of. All authors contributed to the article and approved the submitted version.

## Conflict of Interest

The authors declare that the research was conducted in the absence of any commercial or financial relationships that could be construed as a potential conflict of interest.

## Footnotes

^1^This is not to say that these authors exclusively emphasize (one or both of) these two properties, or that all proposals are uniform in their ambitions, metaphysical commitments and detailed descriptions. Furthermore, there are also models primarily emphasizing different aspects of consciousness, e.g., higher order thought (HOT) theories of consciousness (Brown et al., 2019).

^2^It is less clear whether PCI faithfully reflects variation in richness of experience within (approximately) the same state (Railo et al., 2017; Farnes et al., 2020; Tuominen et al., 2021), or whether it should even be expected to do so, given that ongoing activity is averaged out in the calculation of PCI. However, (Nieminen et al., 2016) suggests that TMS-evoked activity may still index within-state differences in experience, which would also seem to be consistent with IIT's emphasis on possible transitions of the system, over actual activity.

^3^We are not suggesting that richness of experience *equals* signal diversity, we are merely speculating that signal diversity (within certain bounds) may be a useful *correlate* of (brain states favorable to) rich, conscious experience.

^4^In this paper, we use dream/dreaming to mean any conscious experience during sleep, without further requirements.

^5^Reports of dream experience without recall (DEWR) of specific contents could include non-experience (positive response bias), low-quality experiences, and forgotten (or withheld) experiences (Fazekas et al., 2019). In any case, signal diversity for DEWR awakenings should lie (non-strictly) between values for NE and DE awakenings.

^6^To avoid large artifacts/saturation of channels due to movements, recordings were sometimes started after the participant fell asleep, and a few recordings ended up being shorter than 300 s.

^7^More precisely, we used GLMMs with 1-SD (SD = signal diversity) as the response variable, identity link function and gamma-distributed response (see Supplementary Material).
