# Supplementary material for: EEG Signal Diversity Varies With Sleep Stage and Aspects of Dream Experience
Source: Front Psychol. 2021 Apr 23;12:655884. doi: 10.3389/fpsyg.2021.655884 (PMC8102678; doi:10.3389/fpsyg.2021.655884)
Supplement: Supplementary file 1 [file Data_Sheet_1.pdf]

# Supplementary Material

## 1 Supplementary Data

### 1.1 Supplementary Methods

#### 1.1.1 Scoring of sleep stages

For the purpose of sleep scoring and estimation of time of awakening, EEG and EOG channels were temporarily bandpass-filtered between 0.3 - 35 Hz, and the bipolar EMG channel was bandpass-filtered between 1 - 100 Hz (filtering for display only). Bipolar channels F4-TP9, C4-TP9, O2-TP9 and backup channels F3-TP10, C3-TP10, O1-TP10 were temporarily added to aid in identification and assessment of common EEG features such as K-complexes and slow waves (TP9 and TP10 approximating M1 and M2, respectively). A Morlet wavelet time-frequency plot for channel Oz was also added to aid in judgement of posterior dominant alpha-rhythm, low amplitude mixed frequency (LAMF) activity, and slowing of background activity. A marker indicating estimated time of awakening was added to the data during recording and checked offline. The last ten 30 s EEG sleep epochs, relative to time of awakening, were scored in accordance with the AASM Manual for Scoring of Sleep and Associated Events (Berri et al., 2018).

#### 1.1.2 Pre-processing of EEG

Data was pre-processed using the PREP Pipeline (EEG-Clean-Tools) EEGLAB-plugin (Bigdely-Shamlo et al., 2015), including line noise removal, robust average re-referencing, and automatic rejection and interpolation of bad channels. After pre-processing, data was high-pass filtered at 0.75 Hz[1]. Independent component analysis (ICA) was performed using the extended infomax algorithm (Bell and Sejnowski, 1995). To avoid rank deficiency, principal component analysis (PCA) was performed prior to ICA, and the number of principal components to keep was adjusted to account for reduction in rank by average referencing and interpolation of bad channels.

Independent components (ICs) were automatically classified using the IClabel-plugin, which for each IC assigns the probability that the component represents brain, muscle, eye, heart, line nose, channel noise or other sources (Pion-Tonachini et al., 2019). Components were automatically rejected based on their IClabel probabilities if they met any one of the following heuristic criteria:

1. Maximum P(heart) for the recording <u>and</u> P(heart) > 10%,
2. P(brain) < 15 %, or
3. P(brain)/P(artefact) < 2, where P(artefact) is the sum of the probabilities for muscle, eye, heart, line nose and channel noise artefacts.

The cleaned data was low-pass filtered at 40 Hz, and the current source density (surface Laplacian) was calculated by a spherical spline algorithm (Perrin et al., 1989), implemented in the CSD Toolbox

[1] It is often recommended to high-pass filter data at 1 Hz before independent component analysis, but we chose 0.75 Hz to minimize attenuation of slow wave activity in the 0.5-2 Hz band.

(Kayser and Tenke, 2006). The combination of ICA cleaning and spherical-spline Laplacian has been shown to considerably reduce contamination of EEG by muscle artifacts (Fitzgibbon et al., 2015).

### 1.1.3 Generalized linear mixed models

Because we had multiple repeated measurements from the same participant (non-independent and repeated observations), and an unequal number of observations for each condition (unbalanced design), we chose to use mixed effect models for the analysis. In addition to fixed predictors, mixed effects models include random effects that can be used to account for the structure of the data. For example, by including participant ID as a random intercept term, we can account for individual differences in mean response.

The effect of sleep stage on signal diversity was assessed using a generalized linear mixed model with sleep stage as a fixed factor (including intercept), and participant and trial as random intercepts. Sleep trials were coded by unique numbers (implicit nesting) to reflect that trials were nested within participants (no trial occurs for more than one participant). Within each sleep trial, we expect that the observations from different sleep epochs will be correlated to each other, but that the correlation will be smaller the farther apart sleep epochs are from each other. To account for this, and to allow for unequal variances, we specified a heterogeneous first-order auto-regressive residual variance-covariance structure for the sleep epochs (by entering epochs as a repeated effect).

For awakenings in sleep stage NREM2, the effect of experience report (NE, DEWR or DE) on signal diversity of the last sleep epoch before awakening was assessed using a generalized linear mixed model with experience classification as the fixed factor (including intercept) and subject as a random intercept.

Finally, for NREM2 awakenings with reported dream experience (DE), the effect of subjective ratings of experience on a five-point scale from exclusively thought-like to exclusively perceptual on frontal and posterior signal diversity was assessed using a generalized linear mixed model with thought-versus-perceptual rating as a fixed continuous covariate (including intercept) and subject as a random intercept.

We first fitted the models described above as simple linear mixed models, with each of the signal diversity measures (SD) as the response. However, visual inspection of residuals from the resulting models indicated considerable heteroscedasticity of the residuals (the size of the residuals was smaller for higher predicted SD values). This is not very surprising, as signal diversity values are bounded by 1 from above, and some of our data were close to this limit. To account for this, we replaced the dependent variable SD in each of the models by 1 - SD, and instead fitted generalized linear mixed models with gamma-distributed response (right skewed with increasing variance as a function of the mean) and an identity link function, before back-transforming the results. This approach (incorrectly) models SD as unbounded from below, but has the benefit (compared to using other link functions) that the response is still a linear function of the predictor variables. This is important for the analysis of SD as a function of thought-perceptual ratings (modelled as a continuous covariate), for which we hypothesize a linear relationship (Lo and Andrews, 2015).

For the gamma GLMM model of LZC as a function of experience classification of NREM2 dream reports, the confidence interval for the estimated between-participant variation was gigantic (and

obviously not correct for a variable bounded to the unit interval) (**Supplementary Table 1**), which could indicate problems with model fitting (Bolker et al., 2009). The gamma model is not a completely faithful description of the data, since signal diversity values are only bounded on one side. Furthermore, the small sample available for this analysis happened to include two LZC values that would often be classified as extreme (less than Q1 – 3×IQR, i.e. more than 3 times the interquartile range below the first quartile). Excluding only the single most extreme LZC value from the analysis (or winsorizing the two most extreme values to Q1 – 2.6×IQR) resulted in a plausible estimated confidence interval for the between-participant variation, with otherwise broadly similar results. Hence, the problem is likely caused by a pair of "extreme" (but probably perfectly valid) observations in a small sample, which seems to lead to a unrealistic random effect confidence interval for the gamma model.

However, for the categorical factors sleep stage and experience classification, instead of using gamma GLMMs to model 1 - SD as described above, an alternative approach that explicitly bounds signal diversity to the unit interval is to assume that SD follows a beta distribution (only non-zero between 0 and 1), and model the dependency of SD on sleep stage and experience class by generalized linear mixed models with logit (log-odds) link functions. We did this using the R (R Core Team, 2020; RStudio Team, 2020) package glmmTMB (Brooks et al., 2017). For the analysis of SD as a function of sleep stage we modelled the correlation between sleep epochs by an additional random effect (G-side effect) with (homogenous) first order variance-covariance structure, instead of the repeated measures residual effect (R-side effect) used to account for correlation between epochs in the gamma GLMM model in SPSS. Otherwise random effects were the same as for the gamma GLMMs. The significance of sleep stage and experience were assessed by log-likelihood ratio tests comparing these beta models to the corresponding models with only random effects and no fixed predictors. Tests for pairwise contrasts between sleep stages and experience classes were performed on the logit scale. After correcting for multiple comparisons using the adjusted significance levels described below, all statistical tests gave the same results as for the gamma GLMMs. In particular, the beta GLMM and gamma GLMM results for LZC as a function of experience classification of NREM2 dream reports were similar.

### 1.1.4 Controlling family-wise error rate for multiple comparisons

We tested the effect of sleep stage and experience classification on each of the three diversity measures, and the effect of thought-perceptual ratings on both posterior and frontal diversity. In addition to these 12 top level tests, for each signal diversity measure we also performed all 10 pairwise comparisons between the five different sleep stages, and the three pairwise comparisons between the different experience classes (NE, DEWR, DE). To adjust for multiple comparisons, we used the inheritance procedure (Goeman and Finos, 2012), which is a method for family-wise error control (controlling the overall probability of making one or more false discoveries at the specified level $\alpha$, rather than just controlling the expected rate of false discoveries) when testing many hierarchically related hypotheses. Starting with a critical level for significance at $\alpha = 0.05$, this "alpha-wealth" was distributed equally to each of the 12 top level hypothesis[2], yielding an adjusted significance level $\alpha/12 = 0.0042$ for the top level tests. Since sleep stage had significant effects on signal diversity, the "alpha-wealth" from each

[2] Technically, this corresponds to choosing specific, unequal weights for the leaf node hypotheses, which can be freely set before testing, as long as the weight of a "parent" hypothesis is always equal to the sum of the weights for its "children" hypotheses. Since the leaf nodes are not all at the same level of detail in our case, equal leaf weights were not the most natural choice.

of these three tests were further distributed to their respective "heirs", yielding an adjusted significance level of $\alpha/(12\cdot 10) = 0.00042$ for each of the 10 pairwise comparisons between different sleep stages. "Alpha-wealth" from significant pairwise comparisons between sleep stages were further distributed to their "sibling" hypotheses, but this did not lead to any further significant hypotheses[3].

[3] If all hypotheses within a "branch" are significant, “alpha-wealth” can also be inherited by more distant "relatives". A further improvement to the procedure is possible in the case of a (stricter) logical hierarchy of hypotheses.

## 2 Supplementary Figures and Tables

### 2.1 Supplementary Figures

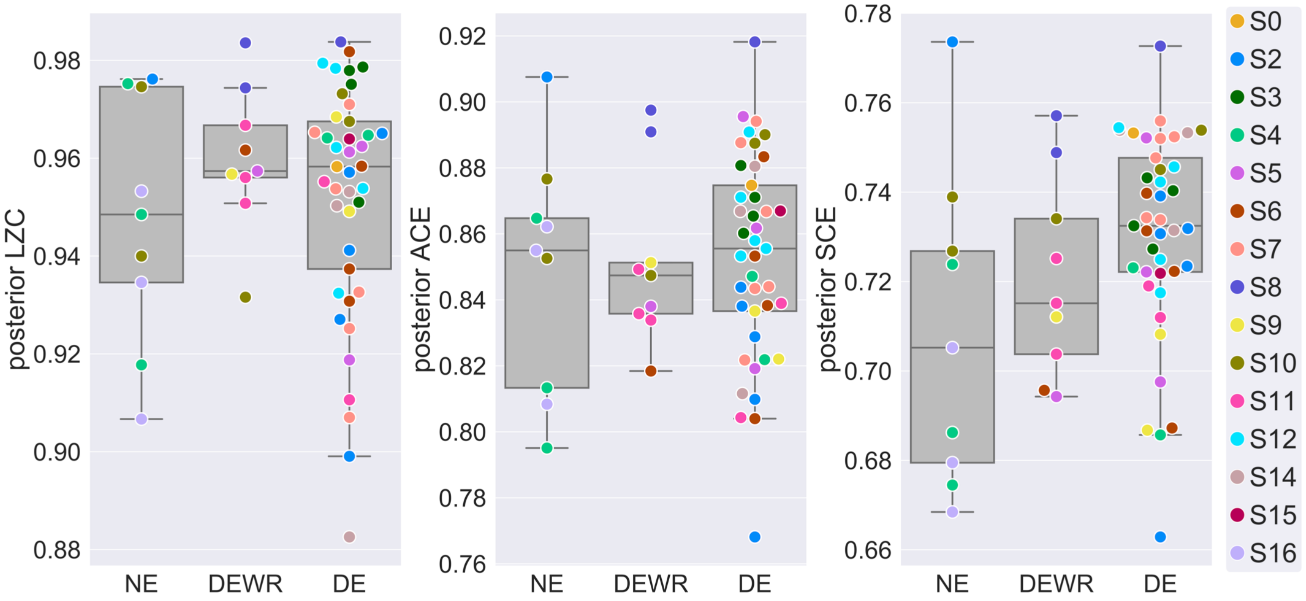


**Supplementary Figure 1. Posterior EEG signal diversity versus dream experience classification.** Mean posterior (see Figure 1a) LZC, ACE and SCE of the last 30 s sleep epoch before NREM2 awakenings, versus experience classification of subsequent dream reports (DE = dream experience, DEWR = dream experience without recall of contents, NE = non-experience). Observations are plotted on top of corresponding boxplots. Participant number (S0, S1, …, S16) is indicated by marker fill color, and observations are displaced slightly along x-axis to avoid overlap.

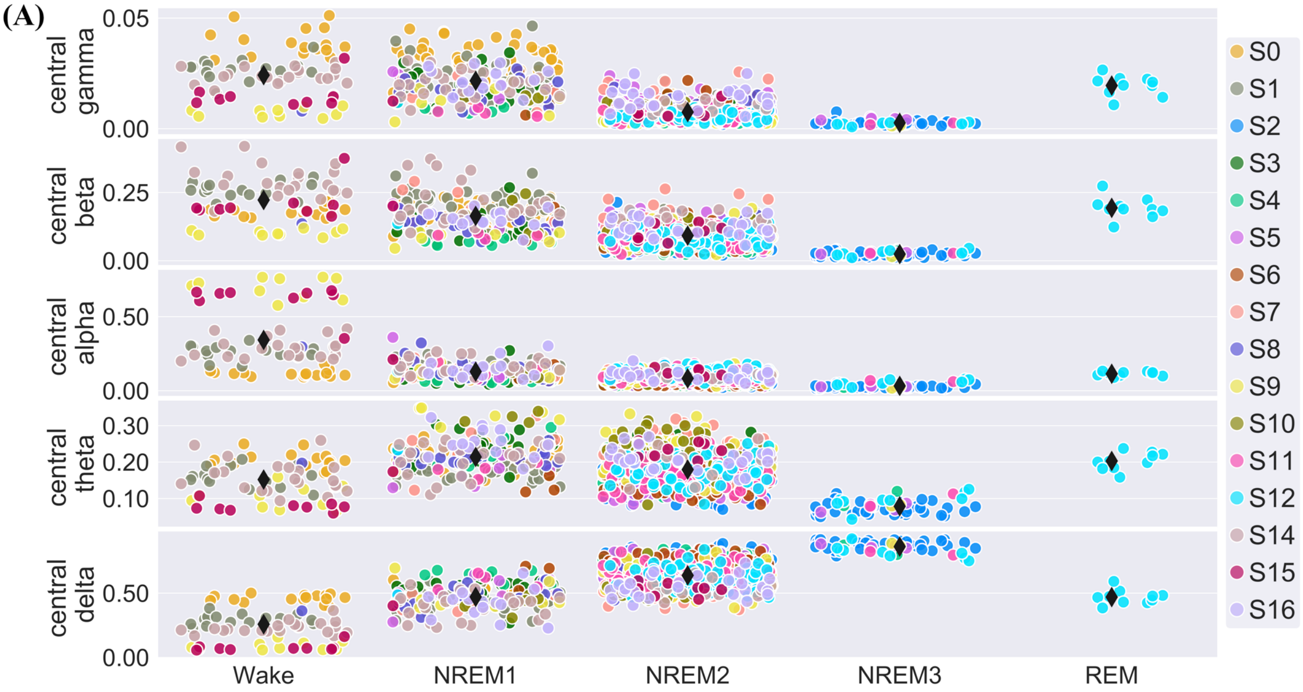


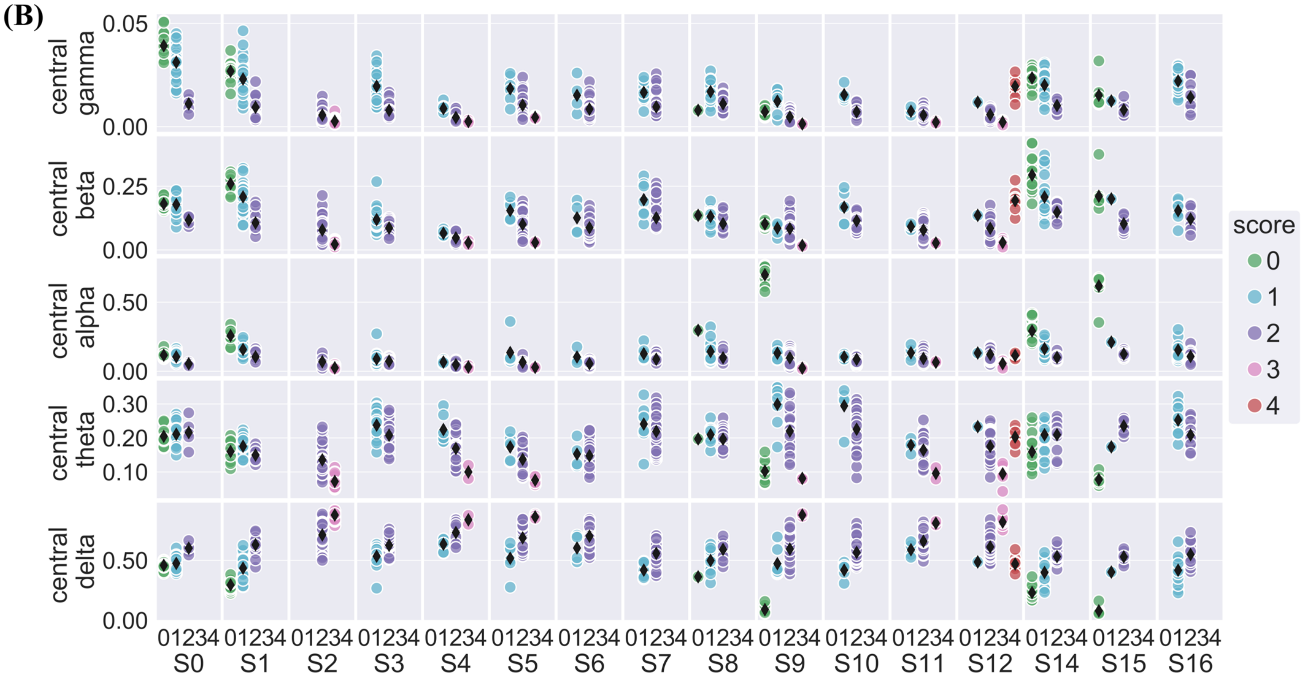


**Supplementary Figure 2. Relative EEG bandpower versus sleep stage. (A)** Power in the delta (0.75-4 Hz), theta (4-8 Hz), alpha (8-12 Hz), beta (12-30 Hz) and gamma (30-40 Hz) bands, relative to total power in the 0.75-40 Hz pass band, for all 30 s sleep epochs versus sleep stage. Power was averaged over central channels and over all the 8 s windows contained within one 30 s sleep epoch. Observations are randomly jittered along x-axis to reduce overlap, and participant number (S0, …, S16) is indicated by marker fill color. Grand mean values for each sleep stage is indicated by black diamonds. **(B)** Power for the 30 s sleep epochs versus sleep stage (0 = W, 1 = NREM1, 2 = NREM2, 3 = NREM3, 4 = REM), plotted separately for each study participant (S0, …, S16). Fill color indicates sleep stage, and diamond markers indicate participant mean values for each stage.

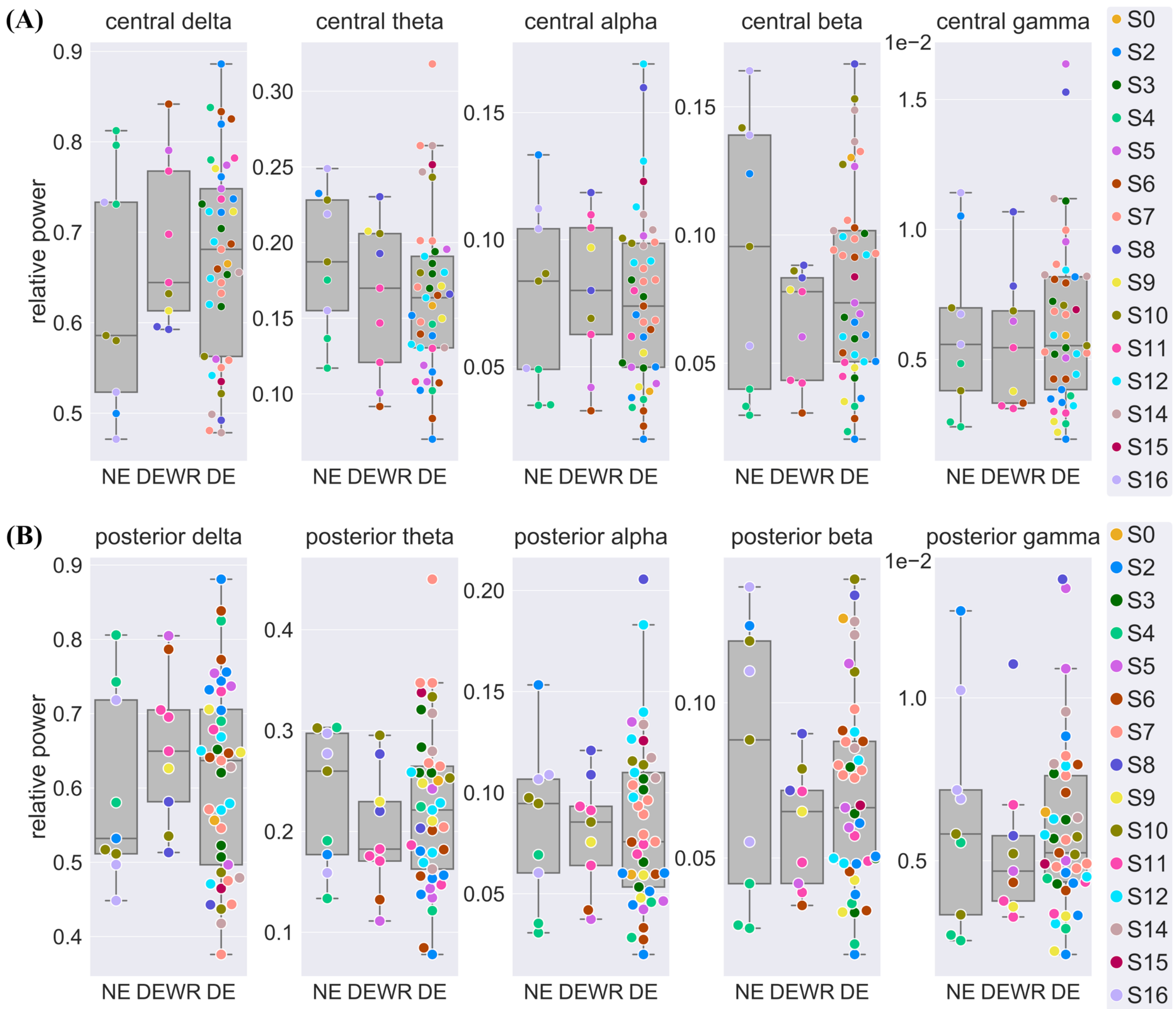


**Supplementary Figure 3. Relative EEG bandpower versus dream experience classification. (A)** Central power in the delta (0.75-4 Hz), theta (4-8 Hz), alpha (8-12 Hz), beta (12-30 Hz) and gamma (30-40 Hz) bands, relative to total power in the 0.75-40 Hz pass band, for the last 30 s sleep epoch before NREM2 awakenings, versus experience classification of subsequent dream reports (DE = dream experience, DEWR = dream experience without recall of contents, NE = non-experience). Power was calculated separately for each channel in the central channel selection and each 8 s window used for calculation of signal diversity measures, then averaged over channels and over all the 8 s windows contained within one 30 s sleep epoch. Observations are plotted on top of corresponding boxplots. Participant number (S0, …, S16) is indicated by marker fill color, and observations are displaced slightly along x-axis to avoid overlap. **(B)** Posterior relative power in the delta (0.75-4 Hz), theta (4-8 Hz), alpha (8-12 Hz), beta (12-30 Hz) and gamma (30-40 Hz) bands (see Figure 1a for illustration of posterior and central channel selections). Otherwise as in panel (A).

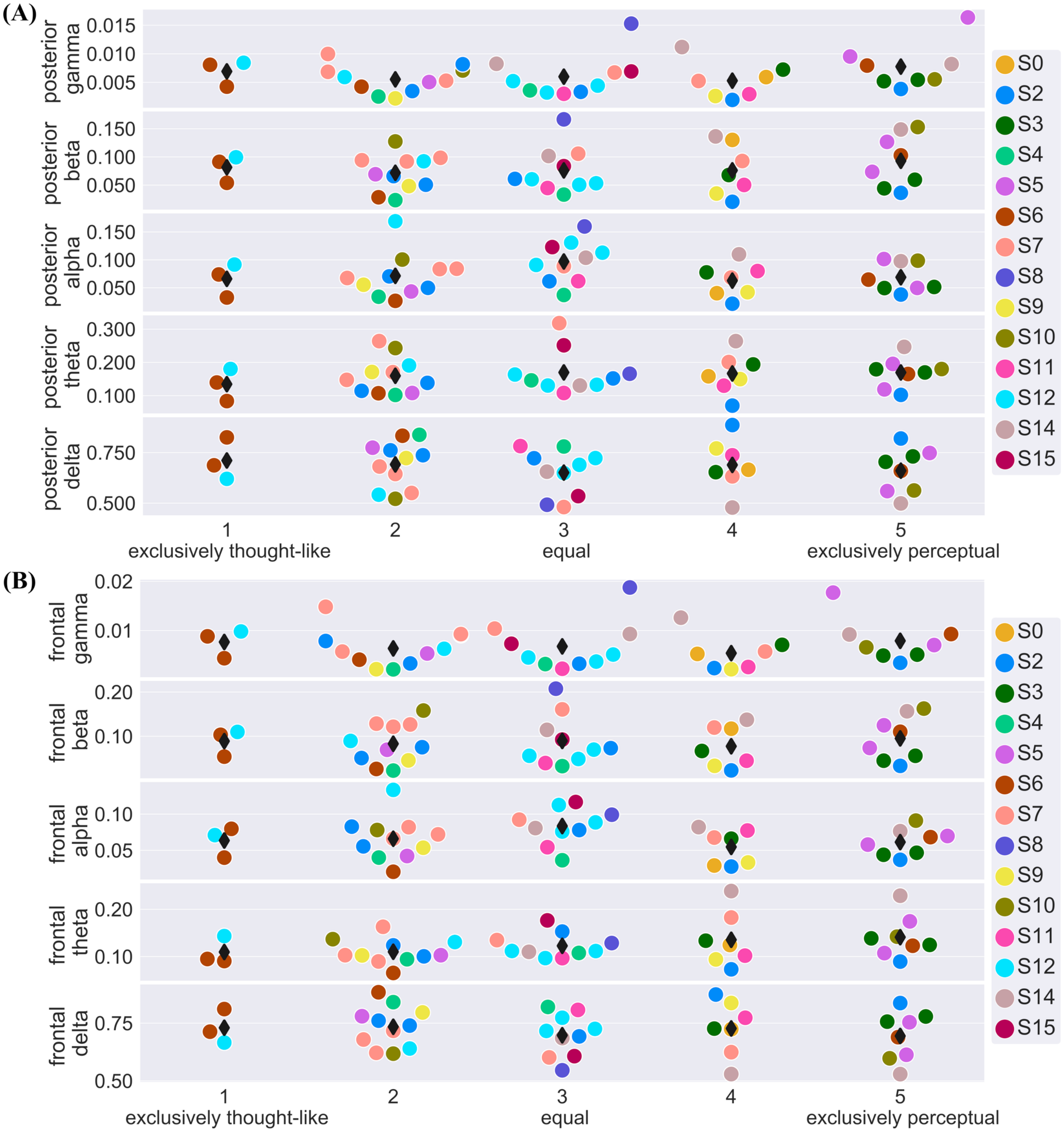


**Supplementary Figure 4. Relative EEG bandpower versus thought-perceptual rating of dream experience. (A)** Power in the delta (0.75-4 Hz), theta (4-8 Hz), alpha (8-12 Hz), beta (12-30 Hz) and gamma (30-40 Hz) bands for posterior channel selection (see Figure 1a), relative to total power in the 0.75-40 Hz pass band, for the last 30 s sleep epoch before NREM2 awakenings with recalled dream experience, versus thought-perceptual ratings of dream contents (1 = exclusively thought-like, 5 = exclusively perceptual). Participant number (S0, …, S16) is indicated by marker fill color, and observations are displaced slightly along x-axis to avoid overlap. **(B)** Frontal (see Figure 1a) EEG bandpower for the last 30 s sleep epoch before NREM2 awakenings with recalled dream experience, versus thought-perceptual ratings of dream content.

## 2.2 Supplementary Tables

**Supplementary Table 1. Signal diversity modelled as a function of sleep stage.** Results for signal diversity modelled as a function of sleep stage, including estimates of between-participant and between-trial variance (back transformed from identity link gamma GLMM for 1–SD).

| **Response** | **Fixed effect** | **df1** | **df2** | **F** | **sig.** | | | | | |
|---|---|---|---|---|---|---|---|---|---|---|
| **LZC** | *sleep stage* | 4 | 139 | 32.5 | <.0001 | | **sig. (pairwise comparison)** | | | |
| | **EMMs** | **estimate** | **std. err.** | **$CI_{lower}$** | **$CI_{upper}$** | | *NREM1* | *NREM2* | *NREM3* | *REM* |
| | *wake* | .976 | .002 | .971 | .981 | | .2068 | <.0001 | <.0001 | .5575 |
| | *NREM1* | .974 | .002 | .970 | .979 | | | <.0001 | <.0001 | .4016 |
| | *NREM2* | .959 | .002 | .955 | .963 | | | | .0049 | .0065 |
| | *NREM3* | .947 | .005 | .937 | .956 | | | | | .0002 |
| | *REM* | .981 | .008 | .965 | .997 | | | | | |
| | **Random effect covariance** | | **estimate** | **std. err.** | **Z** | **sig.** | **$CI_{lower}$** | **$CI_{upper}$** | | |
| | *variance (participant)* | | 4.77e-5 | 2.24e-5 | 2.13 | .0333 | 1.90e-5 | .0001 | | |
| | *variance (participant * trial)* | | 4.35e-5 | 1.17e-5 | 3.71 | .0002 | 2.57e-5 | 7.39e-5 | | |

| **Response** | **Fixed effect** | **df1** | **df2** | **F** | **sig.** | | | | | |
|---|---|---|---|---|---|---|---|---|---|---|
| **ACE** | *sleep stage* | 4 | 169 | 67.7 | <.0001 | | **sig. (pairwise comparison)** | | | |
| | **EMMs** | **estimate** | **std. err.** | **$CI_{lower}$** | **$CI_{upper}$** | | *NREM1* | *NREM2* | *NREM3* | *REM* |
| | *wake* | .906 | .005 | .896 | .916 | | .1295 | <.0001 | <.0001 | .0491 |
| | *NREM1* | .902 | .005 | .893 | .912 | | | <.0001 | <.0001 | .0228 |
| | *NREM2* | .873 | .004 | .864 | .883 | | | | <.0001 | <.0001 |
| | *NREM3* | .828 | .007 | .814 | .843 | | | | | <.0001 |
| | *REM* | .932 | .013 | .906 | .959 | | | | | |
| | **Random effect covariance** | | **estimate** | **std. err.** | **Z** | **sig.** | **$CI_{lower}$** | **$CI_{upper}$** | | |
| | *variance (participant)* | | .0003 | .0001 | 2.45 | .0144 | .0001 | .0006 | | |
| | *variance (participant * trial)* | | .0001 | 2.88e-5 | 3.99 | <.0001 | 7.05e-5 | .0002 | | |

| **Response** | **Fixed effect** | **df1** | **df2** | **F** | **sig.** | | | | | |
|---|---|---|---|---|---|---|---|---|---|---|
| **SCE** | *sleep stage* | 4 | 235 | 37.4 | <.0001 | | **sig. (pairwise comparison)** | | | |
| | **EMMs** | **estimate** | **std. err.** | **$CI_{lower}$** | **$CI_{upper}$** | | *NREM1* | *NREM2* | *NREM3* | *REM* |
| | *wake* | .755 | .005 | .745 | .765 | | .1163 | <.0001 | <.0001 | .5363 |
| | *NREM1* | .751 | .004 | .742 | .760 | | | <.0001 | <.0001 | .3901 |
| | *NREM2* | .740 | .004 | .731 | .749 | | | | <.0001 | .1111 |
| | *NREM3* | .711 | .005 | .700 | .721 | | | | | .0008 |
| | *REM* | .765 | .016 | .734 | .796 | | | | | |
| | **Random effect covariance** | | **estimate** | **std. err.** | **Z** | **sig.** | **$CI_{lower}$** | **$CI_{upper}$** | | |
| | *variance (participant)* | | .0002 | 9.95e-5 | 2.35 | .0188 | .0001 | .0005 | | |
| | *variance (participant * trial)* | | .0002 | 3.44e-5 | 5.04 | <.0001 | .0001 | .0003 | | |

**Supplementary Table 2. NREM2 signal diversity modelled as a function of experience class.** Results for NREM2 signal diversity modelled as a function of experience class, including estimates of between-participant variance (back transformed from identity link gamma GLMM for 1–SD).

| **Response** | **Fixed effect** | **df1** | **df2** | **F** | **sig.** | | | | |
|---|---|---|---|---|---|---|---|---|---|
| **LZC** | *experience class* | 2 | 40 | .516 | .6009 | | | **sig. (pairwise comparison)** | |
| | **EMMs** | **estimate** | **std. err.** | **$CI_{lower}$** | **$CI_{upper}$** | | | *DEWR* | *DE* |
| | *NE* | .956 | .007 | .941 | .971 | | | .6889 | .6679 |
| | *DEWR* | .960 | .007 | .947 | .974 | | | | .3266 |
| | *DE* | .953 | .004 | .945 | .960 | | | | |
| | **Random effect covariance** | | **estimate** | **std. err.** | **Z** | **sig.** | **$CI_{lower}$** | **$CI_{upper}$** | |
| | *variance (participant)* | | 2.42e-6 | 5.41e-5 | .045 | .9643 | 2.45e-25 | 2.40e+13[a] | |

[a] Note the gigantic CI for the estimated between-participant variance, indicating possible problems with model fitting. Excluding/winsorizing the smallest one/two LZC values lead to plausible CI, and gave otherwise similar results, as did a beta GLMM with logit link (Supplementary Methods).

| **Response** | **Fixed effect** | **df1** | **df2** | **F** | **sig.** | | | | |
|---|---|---|---|---|---|---|---|---|---|
| **ACE** | *experience class* | 2 | 56 | .254 | .7766 | | | **sig. (pairwise comparison)** | |
| | **EMMs** | **estimate** | **std. err.** | **$CI_{lower}$** | **$CI_{upper}$** | | | *DEWR* | *DE* |
| | *NE* | .866 | .011 | .843 | .889 | | | .4808 | .6535 |
| | *DEWR* | .856 | .010 | .835 | .877 | | | | .6362 |
| | *DE* | .861 | .006 | .848 | .875 | | | | |
| | **Random effect covariance** | | **estimate** | **std. err.** | **Z** | **sig.** | **$CI_{lower}$** | **$CI_{upper}$** | |
| | *variance (participant)* | | .0004 | .0002 | 1.84 | .0655 | .0001 | .0010 | |

| **Response** | **Fixed effect** | **df1** | **df2** | **F** | **sig.** | | | | |
|---|---|---|---|---|---|---|---|---|---|
| **SCE** | *experience class* | 2 | 54 | 1.03 | .3626 | | | **sig. (pairwise comparison)** | |
| | **EMMs** | **estimate** | **std. err.** | **$CI_{lower}$** | **$CI_{upper}$** | | | *DEWR* | *DE* |
| | *NE* | .725 | .008 | .708 | .741 | | | .6944 | .2224 |
| | *DEWR* | .728 | .007 | .714 | .743 | | | | .3764 |
| | *DE* | .734 | .005 | .724 | .745 | | | | |
| | **Random effect covariance** | | **estimate** | **std. err.** | **Z** | **sig.** | **$CI_{lower}$** | **$CI_{upper}$** | |
| | *variance (participant)* | | .0002 | .0001 | 2.01 | .0446 | 9.32e-5 | .0007 | |

| **Response** | **Fixed effect** | **df1** | **df2** | **F** | **sig.** | | | | |
|---|---|---|---|---|---|---|---|---|---|
| **$LZC_{back}$** | *experience class* | 2 | 51 | .671 | .516 | | | **sig. (pairwise comparison)** | |
| | **EMMs** | **estimate** | **std. err.** | **$CI_{lower}$** | **$CI_{upper}$** | | | *DEWR* | *DE* |
| | *NE* | .948 | .009 | .930 | .966 | | | .2775 | .5650 |
| | *DEWR* | .960 | .007 | .947 | .974 | | | | .3786 |
| | *DE* | .954 | .004 | .946 | .962 | | | | |
| | **Random effect covariance** | | **estimate** | **std. err.** | **Z** | **sig.** | **$CI_{lower}$** | **$CI_{upper}$** | |
| | *variance (participant)* | | 5.12e-5 | 6.60e-5 | .775 | .4381 | 4.09e-6 | .0006 | |

| **Response** | **Fixed effect** | **df1** | **df2** | **F** | **sig.** | | | | |
|---|---|---|---|---|---|---|---|---|---|
| **$ACE_{back}$** | *experience class* | 2 | 56 | .135 | .874 | | | **sig. (pairwise comparison)** | |
| | **EMMs** | **estimate** | **std. err.** | **$CI_{lower}$** | **$CI_{upper}$** | | | *DEWR* | *DE* |
| | *NE* | .855 | .012 | .831 | .879 | | | .7466 | .9516 |
| | *DEWR* | .850 | .011 | .828 | .872 | | | | .6058 |
| | *DE* | .856 | .006 | .842 | .869 | | | | |
| | **Random effect covariance** | | **estimate** | **std. err.** | **Z** | **sig.** | **$CI_{lower}$** | **$CI_{upper}$** | |
| | *variance (participant)* | | .0003 | .0002 | 1.60 | .1092 | 8.95e-5 | .0010 | |

| **Response** | **Fixed effect** | **df1** | **df2** | **F** | **sig.** | | | | |
|---|---|---|---|---|---|---|---|---|---|
| **$SCE_{back}$** | *experience class* | 2 | 55 | .881 | .420 | | | **sig. (pairwise comparison)** | |
| | **EMMs** | **estimate** | **std. err.** | **$CI_{lower}$** | **$CI_{upper}$** | | | *DEWR* | *DE* |
| | *NE* | .721 | .010 | .702 | .741 | | | .8795 | .4160 |
| | *DEWR* | .720 | .009 | .702 | .737 | | | | .2582 |
| | *DE* | .729 | .006 | .717 | .741 | | | | |
| | **Random effect covariance** | | **estimate** | **std. err.** | **Z** | **sig.** | **$CI_{lower}$** | **$CI_{upper}$** | |
| | *variance (participant)* | | .0003 | .0002 | 1.68 | .0923 | 9.11e-5 | .0009 | |

**Supplementary Table 3. Signal diversity for NREM2 awakenings with dream experience, modelled as a function of thought-perceptual rating of dream contents.** Results for NREM2 signal diversity modelled as a function of thought-perceptual rating of dream contents, including estimates of between-participant variance (back transformed from identity link gamma GLMM for 1–SD).

| **Response** | **Fixed effect** | **df1** | **df2** | **F** | **sig.** | **estimate** | **std. err.** | **t** | **$CI_{lower}$** | **$CI_{upper}$** |
|---|---|---|---|---|---|---|---|---|---|---|
| **$LZC_{back}$** | *thought-percept* | 1 | 34 | 9.96 | .0033 | .0076 | .0024 | 3.16 | .0027 | .0124 |
| | *(intercept)* | | | | <.0001 | .9391 | .0075 | 8.10 | .9238 | .9543 |
| | **Random effect covariance** | | **estimate** | **std. err.** | **Z** | **sig.** | **$CI_{lower}$** | **$CI_{upper}$** | | |
| | *variance (participant)* | | .0001 | .0001 | 1.30 | .1950 | 3.01e-5 | .0006 | | |

| **Response** | **Fixed effect** | **df1** | **df2** | **F** | **sig.** | **estimate** | **std. err.** | **t** | **$CI_{lower}$** | **$CI_{upper}$** |
|---|---|---|---|---|---|---|---|---|---|---|
| **$LZC_{front}$** | *thought-percept* | 1 | 31 | 4.45 | .0432 | .0067 | .0032 | 2.11 | .0002 | .0132 |
| | *(intercept)* | | | | <.0001 | .9121 | .0097 | 9.05 | .8924 | .9319 |
| | **Random effect covariance** | | **estimate** | **std. err.** | **Z** | **sig.** | **$CI_{lower}$** | **$CI_{upper}$** | | |
| | *variance (participant)* | | .0003 | .0002 | 1.19 | .2353 | 5.37e-5 | .0015 | | |

| **Response** | **Fixed effect** | **df1** | **df2** | **F** | **sig.** | **estimate** | **std. err.** | **t** | **$CI_{lower}$** | **$CI_{upper}$** |
|---|---|---|---|---|---|---|---|---|---|---|
| **$ACE_{back}$** | *thought-percept* | 1 | 32 | 4.27 | .0472 | .0074 | .0036 | 2.07 | 9.89e-5 | .0147 |
| | *(intercept)* | | | | <.0001 | .8399 | .0107 | 14.9 | .8180 | .8618 |
| | **Random effect covariance** | | **estimate** | **std. err.** | **Z** | **sig.** | **$CI_{lower}$** | **$CI_{upper}$** | | |
| | *variance (participant)* | | .0004 | .0003 | 1.36 | .1725 | 8.95e-5 | .0016 | | |

| **Response** | **Fixed effect** | **df1** | **df2** | **F** | **sig.** | **estimate** | **std. err.** | **t** | **$CI_{lower}$** | **$CI_{upper}$** |
|---|---|---|---|---|---|---|---|---|---|---|
| **$ACE_{front}$** | *thought-percept* | 1 | 29 | 2.31 | .1398 | .0055 | .0036 | 1.52 | .0019 | .0128 |
| | *(intercept)* | | | | <.0001 | .8190 | .0113 | 16.0 | .7960 | .8420 |
| | **Random effect covariance** | | **estimate** | **std. err.** | **Z** | **sig.** | **$CI_{lower}$** | **$CI_{upper}$** | | |
| | *variance (participant)* | | .0006 | .0004 | 1.58 | .1130 | .0002 | .0020 | | |

| **Response** | **Fixed effect** | **df1** | **df2** | **F** | **sig.** | **estimate** | **std. err.** | **t** | **$CI_{lower}$** | **$CI_{upper}$** |
|---|---|---|---|---|---|---|---|---|---|---|
| **$SCE_{back}$** | *thought-percept* | 1 | 34 | .021 | .8864 | .0004 | .0029 | .144 | .0055 | .0063 |
| | *(intercept)* | | | | <.0001 | .7301 | .0080 | 33.7 | .7137 | .7464 |
| | **Random effect covariance** | | **estimate** | **std. err.** | **Z** | **sig.** | **$CI_{lower}$** | **$CI_{upper}$** | | |
| | *variance (participant)* | | .0001 | .0002 | .945 | .3446 | 1.79e-5 | .0011 | | |

| **Response** | **Fixed effect** | **df1** | **df2** | **F** | **sig.** | **estimate** | **std. err.** | **t** | **$CI_{lower}$** | **$CI_{upper}$** |
|---|---|---|---|---|---|---|---|---|---|---|
| **$SCE_{front}$** | *thought-percept* | 1 | 29 | .370 | .5478 | -.0016 | .0026 | -.608 | -.0070 | .0038 |
| | *(intercept)* | | | | <.0001 | .7230 | .0087 | 32.0 | .7053 | .7407 |
| | **Random effect covariance** | | **estimate** | **std. err.** | **Z** | **sig.** | **$CI_{lower}$** | **$CI_{upper}$** | | |
| | *variance (participant)* | | .0004 | .0002 | 1.93 | .0532 | .0002 | .0012 | | |

**Supplementary Table 4. Summary of data used for analysis.** Counts of data used for analysis of how signal diversity varies with sleep stage, experience classification (DE = dream experience, DEWR = dream experience without recall of contents, NE = non-experience) of NREM2 awakening reports and thought-perceptual rating (1 = exclusively thought-like, 5 = exclusively perceptual) of DE NREM2 awakening reports.

| | | Sleep epochs | | | | | Experience class | | | Thought-perceptual rating | | | | |
|---|---|---|---|---|---|---|---|---|---|---|---|---|---|---|
| **Participant** | **Sleep trials** | **Wake** | **NREM1** | **NREM2** | **NREM3** | **REM** | **NE** | **DEWR** | **DE** | **1** | **2** | **3** | **4** | **5** |
| **0** | 7 | 16 | 49 | 5 | 0 | 0 | 0 | 0 | 1 | 0 | 0 | 0 | 1 | 0 |
| **1** | 7 | 15 | 36 | 12 | 0 | 0 | 0 | 0 | 0 | 0 | 0 | 0 | 0 | 0 |
| **2** | 11 | 0 | 0 | 75 | 33 | 0 | 1 | 0 | 5 | 0 | 2 | 1 | 1 | 1 |
| **3** | 6 | 0 | 21 | 39 | 0 | 0 | 0 | 0 | 4 | 0 | 0 | 0 | 1 | 2 |
| **4** | 6 | 0 | 7 | 51 | 2 | 0 | 3 | 0 | 2 | 0 | 1 | 1 | 0 | 0 |
| **5** | 6 | 0 | 6 | 49 | 5 | 0 | 0 | 1 | 3 | 0 | 1 | 0 | 0 | 2 |
| **6** | 6 | 0 | 4 | 56 | 0 | 0 | 0 | 1 | 4 | 2 | 1 | 0 | 0 | 1 |
| **7** | 6 | 0 | 7 | 53 | 0 | 0 | 0 | 0 | 6 | 0 | 3 | 1 | 1 | 0 |
| **8** | 4 | 1 | 19 | 20 | 0 | 0 | 0 | 2 | 1 | 0 | 0 | 1 | 0 | 0 |
| **9** | 6 | 10 | 8 | 40 | 2 | 0 | 0 | 1 | 2 | 0 | 1 | 0 | 1 | 0 |
| **10** | 5 | 0 | 5 | 45 | 0 | 0 | 2 | 1 | 2 | 0 | 1 | 0 | 0 | 1 |
| **11** | 7 | 0 | 4 | 64 | 2 | 0 | 0 | 3 | 2 | 0 | 0 | 1 | 1 | 0 |
| **12** | 8 | 0 | 1 | 60 | 9 | 10 | 0 | 0 | 5 | 1 | 1 | 3 | 0 | 0 |
| **13** | 0 | 0 | 0 | 0 | 0 | 0 | 0 | 0 | 0 | 0 | 0 | 0 | 0 | 0 |
| **14** | 6 | 21 | 24 | 15 | 0 | 0 | 0 | 0 | 3 | 0 | 0 | 1 | 1 | 1 |
| **15** | 2 | 9 | 1 | 9 | 0 | 0 | 0 | 0 | 1 | 0 | 0 | 1 | 0 | 0 |
| **16** | 4 | 0 | 14 | 26 | 0 | 0 | 3 | 0 | 0 | 0 | 0 | 0 | 0 | 0 |

## 3 References


Bell, A. J., and Sejnowski, T. J. (1995). An information-maximization approach to blind separation and blind deconvolution. *Neural Comput.* 7, 1129–1159.

Berri, R. B., Brooks, R., Garnaldo, C. E., Harding, S. M., Lloyd, R. M., Marcus, C. L., et al. (2018). The AASM Manual for the Scoring of Sleep and Associated Events: Rules, Terminology and Technical Specifications, Version 2.5.

Bigdely-Shamlo, N., Mullen, T., Kothe, C., Su, K.-M., and Robbins, K. A. (2015). The PREP pipeline: standardized preprocessing for large-scale EEG analysis. *Front. Neuroinform.* 9, 16.

Bolker, B. M., Brooks, M. E., Clark, C. J., Geange, S. W., Poulsen, J. R., Stevens, M. H. H., et al. (2009). Generalized linear mixed models: a practical guide for ecology and evolution. *Trends Ecol. Evol.* 24, 127–135.

Brooks, M. E., Kristensen, K., van Benthem, K. J., Magnusson, A., Berg, C. W., Nielsen, A., et al. (2017). glmmTMB Balances Speed and Flexibility Among Packages for Zero-inflated Generalized Linear Mixed Modeling. *R J.* 9, 378–400.

Delorme, A., and Makeig, S. (2004). EEGLAB: an open source toolbox for analysis of single-trial EEG dynamics including independent component analysis. *J. Neurosci. Methods* 134, 9–21.

Fitzgibbon, S. P., DeLosAngeles, D., Lewis, T. W., Powers, D. M. W., Whitham, E. M., Willoughby, J. O., et al. (2015). Surface Laplacian of scalp electrical signals and independent component analysis resolve EMG contamination of electroencephalogram. *Int. J. Psychophysiol.* 97, 277–284.

Goeman, J. J., and Finos, L. (2012). The inheritance procedure: multiple testing of tree-structured hypotheses. *Stat. Appl. Genet. Mol. Biol.* 11, Article 11.

Gramfort, A., Luessi, M., Larson, E., Engemann, D. A., Strohmeier, D., Brodbeck, C., et al. (2013). MEG and EEG data analysis with MNE-Python. *Front. Neurosci.* 7, 267.

Kayser, J., and Tenke, C. E. (2006). Principal components analysis of Laplacian waveforms as a generic method for identifying ERP generator patterns: I. Evaluation with auditory oddball tasks. *Clin. Neurophysiol.* 117, 348–368.

Lo, S., and Andrews, S. (2015). To transform or not to transform: using generalized linear mixed models to analyse reaction time data. *Front. Psychol.* 6, 1171.

Perrin, F., Pernier, J., Bertrand, O., and Echallier, J. F. (1989). Spherical splines for scalp potential and current density mapping. *Electroencephalogr. Clin. Neurophysiol.* 72, 184–187.

Pion-Tonachini, L., Kreutz-Delgado, K., and Makeig, S. (2019). ICLabel: An automated electroencephalographic independent component classifier, dataset, and website. *Neuroimage* 198, 181–197.

R Core Team (2020). R: A language and environment for statistical computing. Vienna, Austria: R Foundation for Statistical Computing. Vienna, Austria: R Foundation for Statistical Computing Available at: https://www.R-project.org/.

RStudio Team (2020). RStudio: integrated development for R. Boston, MA: RStudio, PBC. Available at: http://www.rstudio.com.

Schartner, M. M. (2017). On the relation between complex brain activity and consciousness. Available at: http://sro.sussex.ac.uk/67112/ [Accessed December 5, 2018].

Schartner, M., Seth, A., Noirhomme, Q., Boly, M., Bruno, M.-A., Laureys, S., et al. (2015). Complexity of Multi-Dimensional Spontaneous EEG Decreases during Propofol Induced General Anaesthesia. *PLoS One* 10, e0133532.

Siclari, F., Larocque, J. J., Postle, B. R., and Tononi, G. (2013). Assessing sleep consciousness within subjects using a serial awakening paradigm. *Front. Psychol.* 4, 542.
